# The Children's Jubilee Hymn
*From " Hymns for use in the Year of Jubilee 1887." London: Skeffington and Son, 163, Piccadilly.


**Published:** 1887-04-02

**Authors:** Jackson Mason


### THE QUEEN'S JUBILEE.*

The Children's Hymn. By Jackson Mason.

1 Children, up! the lark is soaring,
Now there breaks a gladsome day:
Day, that England scarce has welcomed
Since her kings began their sway;
For the sun of fifty summers
Pours upon our Queen its ray.

2 Cannons boom and flags are flying,
And the steeples shake with joy;
Soon we hear the holy organ,
And with praise our tongues employ;
Soon the psalm ascends for blessings
On our Queen, from man and boy.

3 Let us swell the joyful chorus,
Not alone our voices rise:
Other children seem to join us,
Answering from Paradise;
All for love of her we pray for,
Loyal love, that never dies.

4 Dear the name of our Queen-mother
Is to English boy and maid;
All our joys are hers, our sorrows
Move her tears and ready aid:
O for strong arms to defend her—
Never need she be afraid.

5 Lord of Samuel, Ruth, and David!
On this Jubilee serene
Shed Thy threefold blessing o'er us,
Keep us, for on Thee we lean,
Pure and brave to fight down evil,
True to serve God, Church and Queen. Amen.

* From "Hymns for use in the Year of Jubilee 1887." London: Skeffington and Son, 163, Piccadilly.

